# Lightweight Poly(ε-Caprolactone) Composites with Surface Modified Hollow Glass Microspheres for Use in Rotational Molding: Thermal, Rheological and Mechanical Properties

**DOI:** 10.3390/polym11040624

**Published:** 2019-04-04

**Authors:** Adriano Vignali, Salvatore Iannace, Giulio Falcone, Roberto Utzeri, Paola Stagnaro, Fabio Bertini

**Affiliations:** 1 Istituto per lo Studio delle Macromolecole—CNR, Via A. Corti 12, 20133 Milano, Italy; iannace@ismac.cnr.it; 2 Istituto per lo Studio delle Macromolecole—CNR, Via De Marini 6, 16149 Genova, Italy; giulio.falcone@ge.ismac.cnr.it (G.F.); roberto.utzeri@ge.ismac.cnr.it (R.U.); paola.stagnaro@ge.ismac.cnr.it (P.S.)

**Keywords:** polymer matrix composites, poly(ε-caprolactone), hollow glass microspheres, rotational molding, silanization, morphology, rheological properties, mechanical properties

## Abstract

In this work, novel composites based on poly(ε-caprolactone) (PCL) were prepared and characterized in terms of morphological, thermal, rheological and mechanical properties. Hollow glass microspheres (HGM), alone or surface modified by treatment with (3-aminopropyl)triethoxysilane (APTES) in order to enhance the compatibility between the inorganic particles and the polymer matrix, were used to obtain lightweight composites with improved properties. The silanization treatment implies a good dispersion of filler particles in the matrix and an enhanced filler–polymer adhesion. The addition of HGM to PCL has relevant implications on the rheological and mechanical properties enhancing the stiffness of the material. Furthermore, the presence of HGM strongly interferes with the crystallization behavior and thermo-oxidative degradation of PCL. The increase of PCL crystallization rate was observed as a function of the HGM amount in the composites. Finally, rotational molding tests demonstrated the possibility of successfully producing manufactured goods in PCL and PCL-based composites on both a laboratory and industrial scale.

## 1. Introduction

Due to environmental and sustainability issues induced by the accumulation of plastic waste, the interest in the use of biodegradable polymers is rapidly growing. However, the replacement of petroleum-based polymers for commodities of large-scale applications is still rather limited due to the lower thermal and mechanical properties of biodegradable polymers as well as their relatively high costs [1,2,3]. Among the family of environmentally friendly biodegradable polymers, poly(ε-caprolactone) (PCL) is one of the typically used aliphatic polyesters, being fully biodegradable, biocompatible, and nontoxic to living organisms. PCL also has good flexibility as testified by its high fracture strain. These properties have led PCL to be extensively used since the 1980s in the biomaterials field, particularly for drug delivery and the fabrication of many medical devices. In recent years, the possibility of processing PCL in composite structures, following physical, chemical and/or biological manipulation, has allowed for the implementation of materials with degradation kinetics tailored to fit a specific anatomic site in tissue engineering [4,5]. However, PCL presents the disadvantage of low thermal resistance and heat deflection temperature, as its melting point is only about 60°C. Moreover, the Young’s modulus of PCL is very low, typically 200–400 MPa. These drawbacks have limited its commercial application to some extent. More recently, several PCL-based composites have been prepared in order to improve the mechanical properties of neat PCL. Kai et al. reported on PCL–graphite oxide composites with increased Young’s modulus and tensile strength compared to the neat polymer [6]. Arbelaiz et al. reported interesting tensile and flexural strength data for PCL/flax fiber bundle composites in the presence of maleic anhydride as coupling agent [7]. Kotek et al. investigated the influence of dispersed layered silicate on mechanical and degradation behavior of PCL [8]. Tonelli et al. improved the mechanical properties of PCL by preparing self-reinforced composites made from PCL coalesced from its inclusion compounds formed with host molecules of α-cyclodextrin [9] and urea [10].

In this work, using an alternative approach, we considered to reinforce PCL with hollow glass microspheres (HGM) in order to: (i) obtain lightweight composite materials with enhanced mechanical properties and (ii) widen the application fields of PCL.

HGM consist of an outer shell made of stiff glass filled with an inert gas. This structure provides interesting characteristics, including low density, high stiffness and low thermal and electrical conductivity [11,12]. HGM can be used as a low density filler material ideal for plastic and rubber parts for automotive, aerospace, electrical, leisure and electronic industries. In several studies, HGM have been compounded with polyethylene [13,14,15,16], polypropylene [17,18], thermoplastic polyurethane [19,20], epoxy resin [21,22] and thermoplastic rubber [23], obtaining good results in terms of thermal, dielectric and mechanical properties of the ensuing materials. Lightweight polymer matrix composites with improved mechanical properties can be obtained only when a strong interfacial bonding between the inorganic particles and the polymer matrix is ensured.

Here, the adhesion between the polymer matrix and the inorganic particles was enhanced by modifying the HGM surface with a suitable organosilane, namely (3-aminopropyl)triethoxysilane (APTES), before being embedded in the PCL matrix. The surface modification is based on the condensation reaction which occurs between the ethoxy groups of the silane and the hydroxyls present on the surface of HGM. Thus, a series of dangling chains with amine end groups, connected by Si–O bonds to the microsphere surface, are available for interactions with the polymer matrix [24].

The first aim of this work was to prepare novel PCL-based composites with different content of HGM, both untreated and silanized, and investigate the effect of HGM on their thermal, rheological and mechanical properties. The second aim was to test the processability of the PCL–HGM composites via rotational molding technology to obtain items endowed with a unique and appealing aesthetic appearance.

Rotational molding, also referred to as rotomolding, is a low-shear processing technology that can be used to produce one-piece hollow plastic items ranging from small to large in size, open or closed. This technique allows the preparation of products of any desired shape for various applications like liquid storage tanks, containers, toys and indoor and outdoor furniture [25]. The different grades of polyethylene (PE) currently represent about 85% to 90% of all polymers that are rotationally molded in worldwide production. In recent years, rotomolding has been developed extensively and can nowadays compete with blow molding, injection molding and thermoforming. In many cases, items virtually impossible to fabricate by any other processes can be produced by rotomolding. Using this technique, rigid, resilient hollow bodies are formed from powdered plastic material placed in heated molds, which are rotated simultaneously around two axes perpendicular to each other. The plastic particles make contact and melt in layers on the inner surface of the hot molds until all the powder is fused and the desired end product is obtained. The resultant wall thickness is controlled by the amount of powder placed in the mold. Rotationally molded items are stress-free, except for slight shrinkage forces, because the pieces are produced without any external pressure. The uniformity of wall thickness can be maintained to within ±10%, which is better than that normally obtainable through the blow molding process. Rotational molding also offers other significant advantages when compared with other molding techniques or thermoforming; costs for molds and tooling are relatively low, as well as the resin waste [26]. In recent years, preparation of polymer composites through rotomolding has received the attention of researchers. Yan et al. evaluated the mechanical properties of various micro-sized particulate-reinforced (glass beads, aluminum oxide, silicon carbide) PE produced by the rotational molding process [27]. Planes et al. processed ethylene–propylene copolymer/lamellar graphite nanocomposites by rotational molding. The addition of graphite aims to increase the thermal conductivity of polyolefin and reducing processing cycle time [28]. Gonzalez-Nunez et al. produced PE and agave fiber composites by rotational molding using a simple dry-blending technique. They showed an enhancement of mechanical properties compared with neat PE in terms of tensile, flexural and impact analysis in the presence of 10 wt % fiber concentration [29]. Similar studies on rotomolded polyethylene–agave fiber composites were reported by Robledo-Ortız et al. [30]. They carried out a comparison between different fiber surface treatments to improve the mechanical properties of rotomolded natural fiber composites.

In this work we successfully processed PCL and PCL–HGM composites through the mentioned processing technique by using laboratory and industrial equipment. To the best of our knowledge, this is the first time that rotomolding technology has been used for PCL-based materials.

## 2. Materials and Methods

### 2.1. Materials

The matrix selected for this study was a commercial grade poly(ε-caprolactone) (PCL) purchased from Perstorp under the trade name CAPA 6800. It has a melt flow index (160 °C, 2.16 kg) of 3 g/10 min, a number-average molecular mass of 84,000 g/mol and a density of 1.15 g/cm^3^. The sodium borosilicate hollow glass microspheres (HGM) iM16K, with a density of 0.46 g/cm^3^, average diameter of 20 µm and crush strength of 16,000 psi, were supplied by 3M. APTES ((3-aminopropyl)triethoxysilane) purchased from Sigma-Aldrich, Milan, Italy (purity ≥ 98.5%) was used as the coupling agent.

### 2.2. Hollow Glass Microspheres Surface Treatment

A complete treatment of the HGM surface involves two steps, namely hydroxylation followed by a silanization reaction. In the hydroxylation step, 10 g of HGM were added to NaOH (0.5 mol/L) aqueous solution (400 mL). The resultant suspension was stirred for 1 h at 90 °C in order to create hydroxyl groups on the surface of HGM. Afterwards, the solution was filtered off and the collected HGM were washed with distilled water until the pH of the water reached neutrality. This was followed by vacuum drying at 70 °C for 8 h to eliminate any residual water present on HGM surface. The ensuing hydroxylated HGM were reacted with 2 g APTES dissolved in 200 mL ethanol in the presence of 0.2 g *n*-propylamine as catalyst. This reaction was performed under reflux for 1 h at 70 °C. The functionalized hollow glass microspheres (HGMf) were filtered off and washed with ethanol and water to remove unreacted and/or ungrafted silane. Finally, the material was dried under vacuum at 80 °C for 24 h [31].

### 2.3. Composite Preparation

PCL-based composites were prepared by melt mixing using a Brabender (Duisburg, Germany) electronic plasticorder AEV 153 mixer at 100 °C by applying a rotor speed of 60 rpm for 10 min. During processing, dry nitrogen was continuously purged into the mixing chamber. Neat PCL was treated under identical conditions for reference purposes. Composites containing various amounts of dried HGM alone or functionalized (preheated at 100 °C for 1 h) were prepared Afterwards, the mixed materials were compression molded into 300 μm-thick films by hot-pressing at 100 °C under 60 bar for 3 min, followed by quick cooling to room temperature by cold water.

### 2.4. Methods

To evaluate changes in the functional groups on the HGM surface after chemical treatments, Fourier transform infrared spectroscopy (FTIR) was performed. The spectra were acquired in attenuated total reflectance (ATR) mode in the spectral range of 4000–450 cm^−1^ using Perkin Elmer Waltham, MA, USA) Spectrum Two spectrophotometer equipped with a diamond ATR crystal. 

The morphology of the samples at the micro-scale level was investigated using a benchtop scanning electron microscope (SEM) Hitachi (Tokyo, Japan) TM3000. The inspected surfaces of the composites were obtained through a brittle fracture in liquid nitrogen of compression-molded samples. A thin layer of gold was sputtered onto the fracture surfaces before observation.

Thermogravimetric analysis (TGA) were performed on a Perkin Elmer (Waltham, MA, USA) TGA 7 instrument at a scan rate of 20 °C/min under a dry air atmosphere (flow 30 mL/min). TGA and derivate thermogravimetry (DTG) curves were recorded from 50 °C up to 750 °C.

The molecular characterization was carried out by a conventional size exclusion chromatography (SEC) system calibrated toward polystyrene standards, using tetrahydrofuran (THF) as the mobile phase, 220.7 µL injection volume, 2 mg/mL concentration and 35 °C. The integrated chromatographic system Waters (Milford, MA, USA) GPCV2000 consisted of a degasser, pump and injector and a 2414 differential refractometer used as a concentration detector. The column set was composed of three columns (Polypore, Oligopore and 100 Å, 5 μm of particle size) from Polymer Laboratories. 

Calorimetric analysis was carried out under nitrogen flow using a Perkin Elmer (Waltham, MA, USA) DSC 8000. The samples were heated from −20 °C to 100 °C at a rate of 20 °C/min and kept at 100 °C for 3 min to erase previous thermal history. Then they were cooled to −20 °C at 10 °C/min and subsequently heated at the same rate up to 100 °C. The degree of crystallinity of the PCL matrix, χ_c_, was evaluated by applying the following equation:

(1) $$\chi_{c}=\frac{\Delta H_{m}}{w_{p}\cdot \Delta H^{0}}\cdot 100$$

where *w*_p_ is the weight fraction of PCL in the examined samples, Δ*H*_m_ and Δ*H*^0^ are, respectively, the measured melting enthalpy and the enthalpy of fusion of the 100% crystalline PCL, taken as 139.1 J/g [32].

To estimate the rheological behavior of the composites, a rotational rheometer TA Instruments (New Castle, DE, USA) AR 2000 operating in stress-controlled conditions with circular plates was used. Frequency sweep tests in the range between 0.04 rad/s and 628.3 rad/s were performed at 100 °C and strain amplitude 1% using parallel plates with a diameter of 25 mm and a gap of 0.5 mm.

Uniaxial tensile tests were carried out using a Zwick–Roell (Ulm, Germany) Z010 dynamometer with a load cell of 50 N. Two deformation rates were used: 5 mm/min for small strain values (0–2%), to better evaluate the tensile modulus, and 15 mm/min from 2% onwards, to follow the mechanical behavior until the breaking. Dumbbell-shaped specimens with an overall length of 75 mm, a gauge length of 25 mm and a width of narrow section of 4 mm were cut from compression molded sheets (thickness about 0.3 mm). At least five specimens were tested for each individual sample.

### 2.5. Rotational Molding Tests

**Figure 1:**
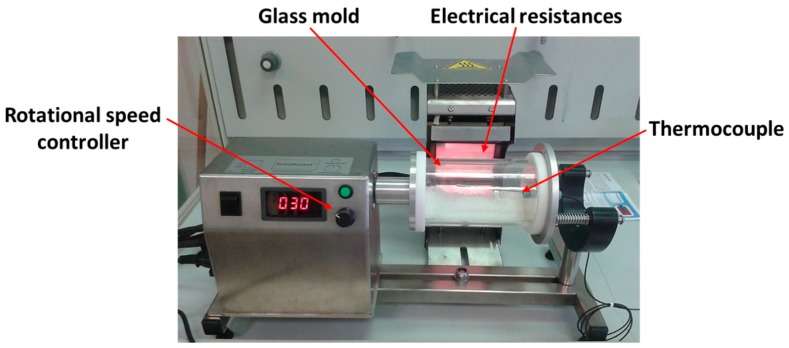
Uniaxial benchtop rotomachine.

Rotational molding experiments were performed using a laboratory-scale machine RotoRocket Science from 493K (Ballyclare, North Ireland, UK) (Figure 1). It consists of a cylindrical glass mold with reduced dimensions (length of 15 cm, diameter of 9 cm), which can rotate only uniaxially. Mold heating occurs through electrical resistances side-placed to the mold, whereas cooling occurs with air. The internal air temperature (IAT) is monitored in real time during the process with a thermocouple connected to a computer. Before loading the material, a demolding agent was applied to the internal surface of the mold. In all cases, an amount of 50 g of material (a mixture of HGM and PCL powders) was loaded into the mold to produce parts with an approximate wall thickness of 2 mm. The charged mold was then closed, mounted on the rotating arm and heated by the resistances. The mold was kept rotating for about 30 min at a rotational speed of 8 rpm for neat PCL and 20 rpm for the composites. After the heating cycle, forced air for cooling was used until the IAT dropped to 25 °C, and the part was then demolded.

## 3. Results and Discussion

### 3.1. Characterization of Hollow Glass Microspheres

**Scheme 1:**
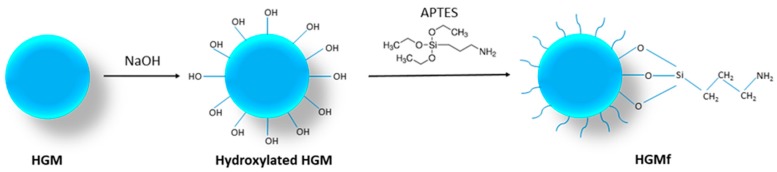
Reaction path for the functionalization of hollow glass microspheres (HGM) particle surface. APTES = (3-aminopropyl)triethoxysilane; HGMf = functionalized HGM.

The silyl coupling agent was anchored to the HGM by a reaction between the ethoxy groups borne by the APTES organosilane with the hydroxyls present on the external surface of the microspheres previously treated with NaOH, as shown in Scheme 1.

**Figure 2:**
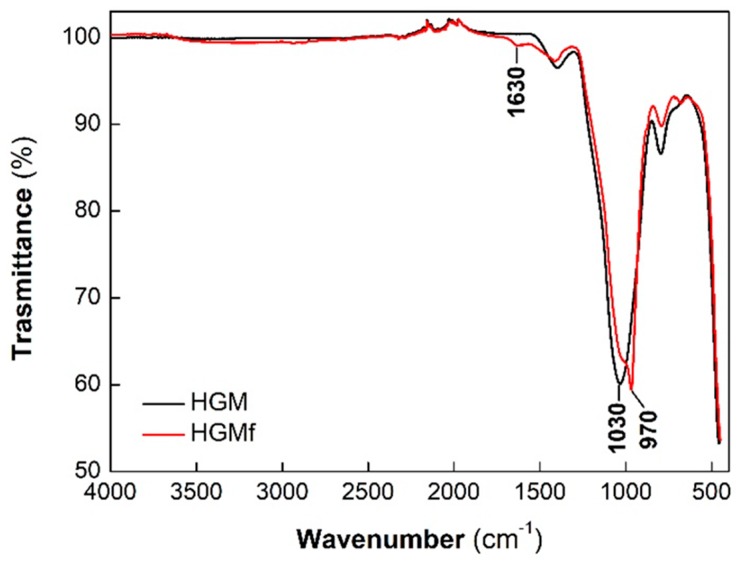
FTIR spectra of naked HGM and HGM functionalized with APTES.

Figure 2 shows the ATR-FTIR spectra of HGM alone and functionalized (HGMf) using the abovementioned method.

The FTIR curves of both HGM show a characteristic peak between 1030 cm^−1^ and 970 cm^−1^ corresponding to the stretching of Si–O–Si bond. The FTIR spectrum of HGMf shows a peak at 1630 cm^−1^ corresponding to the –NH2 scissoring of primary amine groups. This characteristic peak confirms the anchoring of amine groups borne by APTES moieties onto the previously hydroxylated HGM surface.

**Figure 3:**
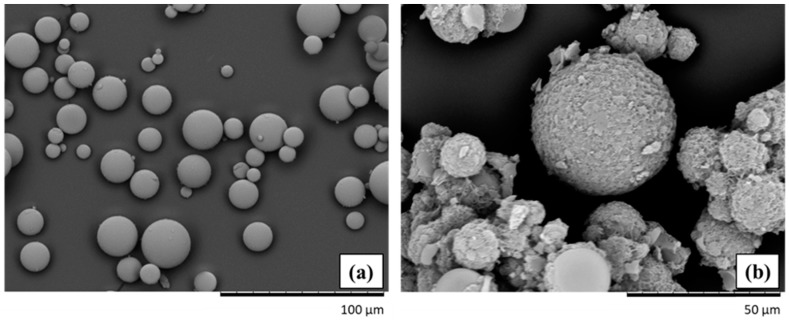
Scanning electron microscopy (SEM) image of HGM alone (**a**) and after surface modification with APTES (**b**).

This result is confirmed by the different morphologies of the hollow glass microspheres observed before and after the surface treatment. Figure 3a shows a SEM micrograph of unmodified HGM. The glass microspheres present a clean, smooth surface, which does not promote interfacial adhesion with the polymer matrix. In Figure 3b, taken at higher magnification, a dense layer of organic material, which covers the glass surface, is observed due to the formation of amine functionalized glass microspheres, HGMf [31,33].

**Figure 4:**
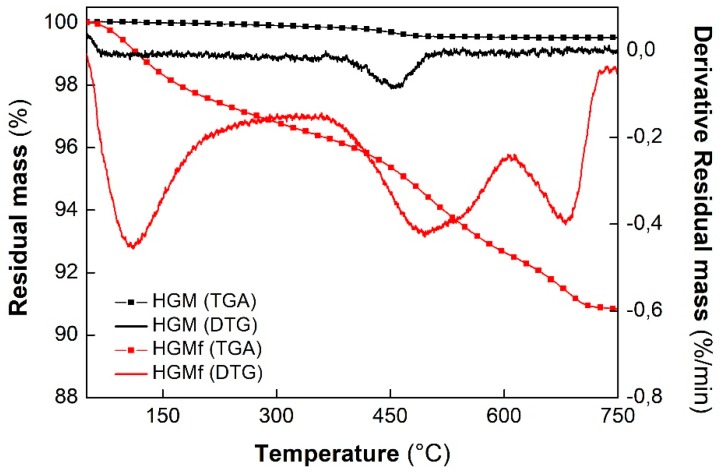
Thermogravimetric analysis (TGA) and derivate thermogravimetry (DTG) curves of naked HGM (black line) and silanized HGMf (red line).

The immobilization of the coupling agent on HGM surface was also proven by TGA, as shown in Figure 4. The TGA curve of the naked HGM exhibits a very small mass loss (about 0.5%) in the temperature range of 50–750 °C. This indicates a low amount of hydroxyl groups present on the glass surface, with consequently very low mass losses due to: (i) physically adsorbed water (up to 200 °C) and (ii) dehydroxylation by condensation of silanols (DTG peak centered at about 450 °C). The thermogram of the HGMf sample shows a broad degradation region between 50 °C and 700 °C, typical of APTES functionalized nanoparticles [34,35].

The DTG profile, constituted by successive decomposition events, indicates that the organic products are released in steps that reflect the different involved mechanisms. The first mass loss occurring at lower temperature (100–200 °C) is associated with the evolution of the water physically adsorbed on the surface (indeed, before silanization, a number of hydroxyl groups have been introduced onto the surface of HGM by treatment with NaOH). The second decomposition stage takes place in the temperature range of 350–700 °C and mainly arises from the thermal oxidative degradation of APTES moieties anchored onto the surface of HGM; it is characterized by a remarkable mass loss (5.5%) with two DTG maxima at 480 °C and 680 °C.

### 3.2. Composite Characterization and Properties

#### 3.2.1. Molecular Characterization and Morphology

Table 1 presents the molecular weight characteristics, i.e., the average molecular weight *M_w_* and the molecular weight distribution *M_w_/M_n_*, of the samples prepared by melt mixing, as well as of the unprocessed PCL. All the materials show similar *M_w_* and *M_w_*/*M_n_* values, indicating that the adopted mixing conditions (which are more severe than those the material will encounter in the rotomolding process) do not induce thermo-mechanical degradation of the polymer chains during the melt processing.

The theoretical density of the investigated materials can be calculated using the following equation:

(2) $${\rho =w}_{PCL}\rho_{PCL}{+w}_{HGM}\rho_{HGM}$$

where *w* is the weight fraction and *ρ* is the density. 

**Figure 5:**
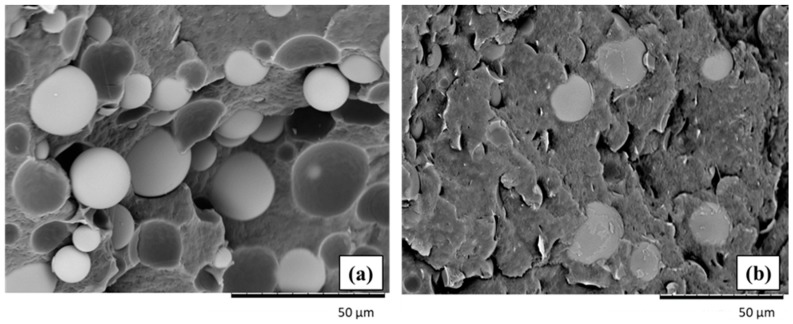
SEM image of PCL composites with 10% of HGM alone (**a**) and after surface modification with APTES (**b**).

HGM are 60% lighter than PCL in agreement with the densities reported by the suppliers. Thus, by increasing the weight fraction of HGM, there is a monotonous decrease in the density of PCL–HGM composites (Table 1). A significant density reduction of 12% and 15% with the respect to neat PCL is observed with the addition of 20 wt % and 25 wt % of HGM, respectively. These results highlight that filling PCL with HGM can lead to lightweight composites. Although the densities of the composites presented in this work are calculated, we believe on their reliability. Indeed, in some papers, the experimental densities calculated for PE-based composites filled with HGM resulted in being very similar to the theoretical value, when HGM contents range from 10 wt % to 30 wt % [16,33]. Moreover, as shown by the SEM micrographs, almost all of the HGM remain uncrushed after the mixing and the subsequent compression molding step (Figure 5). Therefore, the actual density of samples should not be significantly affected by processing, remaining very close to the calculated value.

The morphologies of representative samples are shown in Figure 5. The composites containing the HGM alone exhibit a uniform distribution of the particles but poor adhesion to the polymer matrix, as evidenced by the spherical cavities and the free spheres observable on the surface obtained after fragile fracture in liquid nitrogen (Figure 5a). A better adhesion was achieved in the case of silanized HGMf, as shown in Figure 5b, where cavities are practically absent and the glass spheres are made wet by the PCL matrix and well adhered to it.

#### 3.2.2. Thermal Behavior

**Figure 6:**
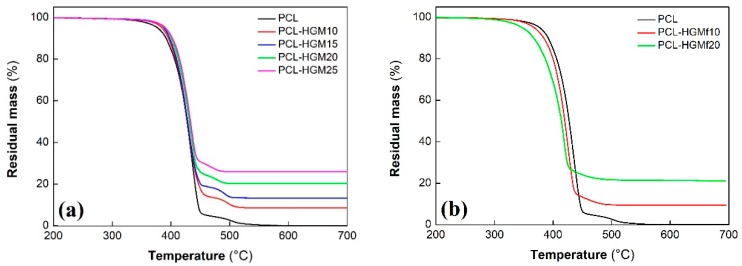
TGA curves in air atmosphere of PCL and PCL–HGM composites (**a**), PCL and PCL–HGMf composites (**b**).

The effect of HGM on the thermal stability and degradation of the composites was investigated by TGA experiments, carried out under an oxidative atmosphere (Figure 6). Table 2 summarizes the TGA experimental data including the temperature at which the initial 5% mass loss occurs (*T*_5%_), the temperature corresponding to 50% mass loss (*T*_50%_) and the residue amount (R) registered at the end of the thermal cycle.

The thermo-oxidative degradation of neat PCL mostly takes place during the first step from 300 °C to 450 °C; this is followed by a minor mass loss stage (about 4%) from 450 °C to 530 °C due to the oxidation process of the carbonaceous residue. The presence of HGM gives rise to an overall increase in the thermal stability of the composites compared to the reference PCL (Figure 6a). The *T*_5%_ value of the PCL-based composites enhances progressively with the increase of HGM content up to an improvement of 15 °C obtained for PCL–HGM25 compared to the PCL sample.

Analogously, although to a minor extent, the *T*_50%_ value enhances with the HGM content, going from 426 °C for neat PCL to 432 °C for PCL–HGM25. The behavior of HGM as a promoter of thermal stability was also observed by Li et al. in the case of poly(butylene succinate)–HGM composites and attributed to the physical barrier effect exerted by the HGM [36]. The barrier effect concerns the diffusion of the oxygen from the external gas phase to the polymer matrix bulk and, at the same time, the opposite out-diffusion of the volatile decomposition products [37]. Conversely, the thermograms of composites filled with HGMf (Figure 6b) exhibit a shift towards lower temperatures because of the presence of less stable APTES moieties anchored onto the surface of HGM. This behavior involves a reduction of thermo-oxidative stability and is more evident for PCL–HGMf20. The residue values reported in Table 3 confirm that, for each sample, the actual content of filler is consistent with the nominal amount employed.

**Figure 7:**
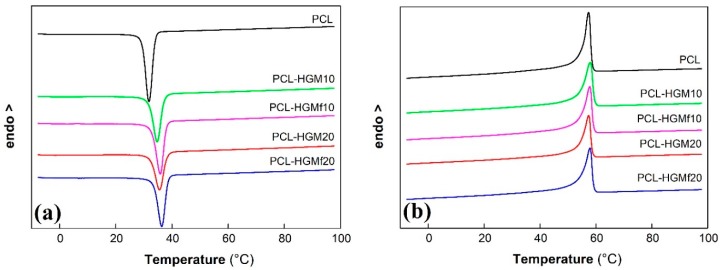
DSC cooling scans (**a**) and DSC successive heating scans (**b**) of PCL and selected PCL-based composites.

As is well known, the addition of fillers may influence the crystallization and melting behavior of the polymer matrix, thus affecting the mechanical properties of the materials. The effect of HGM on the thermal properties of PCL-based composites was investigated by DSC scans carried out in non-isothermal conditions. The values of crystallization temperature (*T*_c_), crystallization enthalpy (Δ*H*_c_), melting temperature (*T*_m_), melting enthalpy (Δ*H*_m_) and crystallinity (χ_c_) are listed in Table 3. Figure 7a,b show the cooling behavior of neat PCL and PCL-HGM composites starting from homogeneous melt conditions and the successive heating curves, respectively. As a whole, compared to neat PCL, the prepared PCL-HGM composites show higher crystallization peak temperature (*T*_c_). By increasing the HGM loading, the corresponding *T*_c_ continues to increase suggesting an important nucleating effect of HGM [36,38]. Moreover, *T*_c_ enhancement is even more pronounced using silanized HGMf.

The measured crystallization and melting enthalpies of composites decrease by increasing the HGM content, whereas the χ_c_ of the filled materials remain substantially unchanged and similar to that of neat PCL (about 38%).

As shown in Figure 7b, on heating, all PCL-based materials present a similar narrow symmetrical endotherm with *T*_m_ at 57 °C, which is the characteristic melting point of PCL. Therefore, the presence of HGM filler, alone or silanized, has no effect on PCL melting behavior.

A comprehensive study on the nucleating effect of HGM on the PCL crystallization will be presented in a following work.

#### 3.2.3. Rheological Properties and Mechanical Behavior

**Figure 8:**
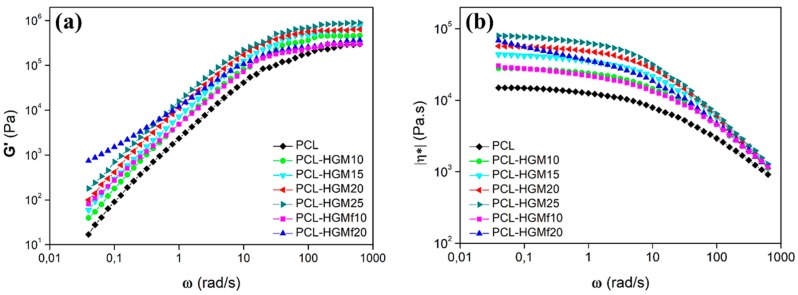
Storage modulus (G′) (**a**) and complex viscosity (η*) (**b**) vs. angular frequency (ω) for neat PCL and PCL-based composites.

To evaluate the influence of the filler content and type on the main viscoelastic parameters, the storage modulus (G′), the loss modulus (G″) and the complex viscosity (η*) of PCL and PCL-based composites were measured at 100 °C as a function of the frequency (Figure 8).

It is evident that the elastic modulus (G′) of the composites increases with the increase of HGM content. In fact, the presence of the inorganic filler leads to an enhancement of the moduli, especially G′, over the whole frequency range investigated (Figure 8a). This result indicates that the polymer relaxations are effectively restrained by the presence of HGM. Therefore, the incorporation of HGM improves the stiffness of PCL. In particular, for the composite PCL–HGMf20, a shift of G′ towards higher values (about two and one order of magnitude compared to neat PCL and PCL filled with 20 wt % of untreated HGM, respectively) is observed between 0.04 rad/s and 0.1 rad/s. In this range, a plateau appears that highlights a solid-like behavior and strong filler–matrix interactions, implying the formation of a percolated filler network with a marked elastic connotation. The complex viscosity (η*) of the composites increases with respect to neat PCL accordingly with the enhancement of both the viscoelastic moduli (Figure 8b). It is clearly observed that, in the frequency range studied, the complex viscosity of neat PCL and of all the filled systems shows a shear thinning effect, which is ascribed to the reduction of entanglement density under the influence of high shear stress. Furthermore, the composites exhibit a significant increase in viscosity as a function of HGM content. This result can be attributed to the interactions established between the surface of HGM and polymer melt. Finally, it is worth noting the complex viscosity of HGMf composites increases compared to HGM composites at the same filler content due to enhanced filler–matrix interactions promoted by the silane coupling agent.

**Figure 9:**
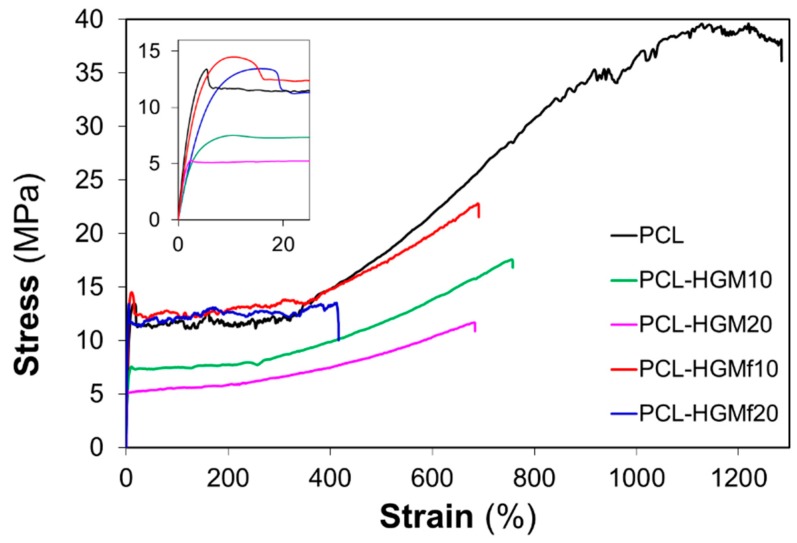
Stress–strain curves of PCL and selected PCL-based composites during monotonic tensile deformation.

The effect of the fillers on the mechanical behavior of PCL-based materials was also evaluated carrying out tensile tests by uniaxial stretching until failure. The stress–strain curves exhibit yielding phenomena to different extents and strain hardening at high deformation (Figure 9). In Table 4, the principal mechanical properties of the samples, namely Young’s modulus (E), yield stress (σ_y_) and elongation at break (ε_r_), are summarized.

PCL exhibits a marked strain hardening, determinant for the higher ductility of the neat polymer compared to the composites. The presence of hollow glass microspheres alone leads to an increase of the tensile modulus accompanied by a reduction of the tensile strength and of the elongation at break. This enhancement of the modulus is more evident at higher HGM concentrations, while the decrease of the tensile strength is rather similar for untreated HGM with a concentration of 15 wt %, 20 wt % and 25 wt %.

The PCL–HGMf composites exhibit an increase in both the tensile modulus and tensile strength compared to the neat polymer and all composites containing unmodified microspheres. This behavior is due to tendency of –NH2 groups of functionalized HGMf to chemically interact with the PCL macromolecules, implying a better adhesion between the surface modified filler and the polymer matrix. In detail, the Young’s modulus and the yield strength improve respectively by about 61% and 10% in presence of 10 wt % silanized HGMf. Furthermore, these properties increase respectively by about 113% and 8% with the addition of 20 wt % HGMf compared to the neat polymer matrix. From Table 4, it is evident that the introduction of 10 wt % HGM causes a decrease of about 45% in elongation at break with a consequent reduction in ductility if compared to neat PCL. This negative trend continues with increases in the HGM content in the composite up to a maximum of 56% in the presence of 25 wt % HGM. A similar trend is observed for the PCL–HGMf series. It is worthy to note that PCL–HGMf20 exhibits the lowest value of elongation at break with 70% decrease compared to PCL.

### 3.3. Rotational Molding Tests

**Figure 10:**
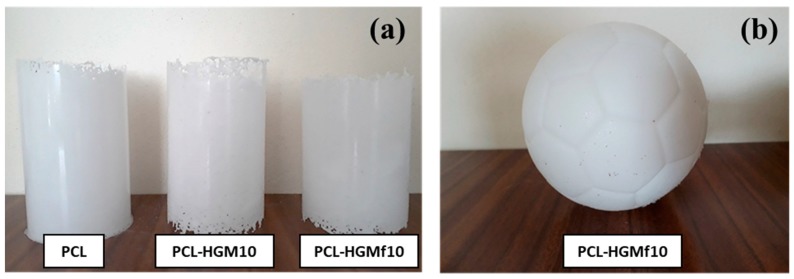
Rotomolding prototypes produced using lab equipment (**a**) and industrial machine (**b**).

Several rotomolded parts in neat PCL and composites based on PCL filled with HGM as such or surface modified with APTES were prepared successfully through the above mentioned uniaxial benchtop rotomachine (see Figure 1), demonstrating their potential use in industrial rotomolding technology (Figure 10a). For such experiments, on the basis of the rheological behavior observed for composites prepared by melt blending, a content of 10 wt % HGM and HGMf was selected as the best filler concentration. This comes from the solid-like behavior observed for the composite PCL–HGMf20 (Figure 8a), which affects the melting flow of the material during processing at low shear rate (Figure 8b). Therefore, an excessive increase of the filler content would presumably complicate the rotational molding of the composites.

**Figure 11:**
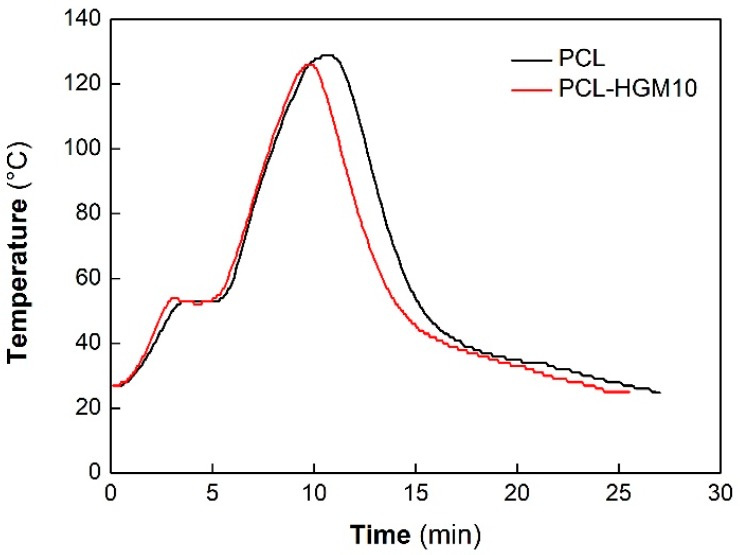
Internal air temperature as a function of time during rotomolding tests.

The most striking result for neat PCL and PCL charged with HGMf was the similar behavior observed during the rotomolding experiments. In fact, all the materials were processed at the same temperature as shown in Figure 11, where the recorded internal air temperature (IAT) is reported as a function of time for neat PCL and PCL–HGM10. The resulting thermograms present a plateau at about 50 °C in correspondence with the point where PCL powder begins melting. At the end of this plateau, when the powder has completely melted, the IAT rises again until about 125 °C for each material. Crystallization is promoted by cooling gradually the mold. During cooling at around 40 °C, a slight change in the slope of the IAT thermograms is observed due to the detachment of the material from the mold surface and the subsequent formation of an insulating layer of air in between them. Once the demolding temperature (25 °C) is reached, the final product is removed from the mold. The entire rotomolding process for both materials required about 25 min for completion. Finally, few PCL-based formulations were selected and used to successfully produce lightweight prototypes with good surface finishing (Figure 10b) at an industrial scale by means of a biaxially rotating machine.

It is necessary to remark that this is an important goal from an industrial point of view. Indeed, the possibility of rotomolding PCL-based materials which need very low processing temperatures (e.g., with respect to polyethylene-based materials) would not only lead to significant energy savings, but would also allow for the use of fiberglass reinforced epoxy molds, which is much less expensive compared to the normally-used metal molds.

## 4. Conclusions

In the present work, we prepared PCL-based composites by melt blending consisting of a PCL matrix filled with hollow glass microspheres. We evaluated the influence of a silanization treatment on the HGM, comparing the properties of composites containing both untreated HGM and surface functionalized HGMf. The morphological analysis showed good distribution/dispersion of the glass particles into the polymer matrix in all cases while a good filler–matrix adhesion was observed only in the case of silanized HGMf. Thermal characterization highlighted an improved crystallization rate and thermal stability of composites compared to neat PCL. A pseudo solid-like behavior, characterized by a plateau of G’ at low frequencies, in the presence of 20 wt % silanized HGMf was observed through rheological measurements, highlighting stronger filler–matrix interactions. The addition of HGM affected the mechanical properties, leading to a significant enhancement in material stiffness. In particular, tensile tests showed a high increase in Young’s modulus for all investigated composites compared to neat PCL. This behavior was combined with a decrease of elongation at break with a consequent reduction in material ductility. Nevertheless, the composites filled with the modified HGMf, characterized by an enhancement of the tensile strength, pointed out the important role played by the chosen silane coupling agent (APTES) in improving filler–matrix adhesion. This is a remarkable result, considering that the addition of 20 wt % HGM implies a decrease of density by about 12% compared to neat PCL. In the present case, unlike other typical lightweight polymeric materials such as foams, lowering the composites density is not accompanied by reduced mechanical properties. Finally, PCL and HGM composites were proven to be successfully processed by rotational molding technique, both with laboratory and industrial equipment. The obtained results validate the incorporation of hollow glass microspheres as an interesting solution to improve the properties of PCL, specifically leading to mechanical reinforcement, weight reduction and good aesthetic appearance. This extends PCL applications in general and in particular, relative to the focus of this work, to items which can be easily manufactured through rotational molding.

## Figures and Tables

**Table 1:** Composition by weight and molecular data of the investigated materials.

Sample	PCL/HGM/HGMf	*M_w_* (kg/mol)	*M_w_*/*M_n_*	Theoretical Density (g/cm^3^)
PCL unprocessed	100/0/0	177.1	2.1	1.15
PCL	100/0/0	177.3	2.0	1.15
PCL–HGM10	90/10/0	187.8	2.0	1.08
PCL–HGM15	85/15/0	183.9	2.0	1.05
PCL–HGM20	80/20/0	182.4	2.0	1.01
PCL–HGM25	75/25/0	181.4	2.0	0.98
PCL–HGMf10	90/0/10	185.0	2.1	1.08 ^1^
PCL–HGMf20	80/0/20	180.8	2.1	1.01 ^1^

^1^ Calculated using the density of untreated HGM. *M_w_* = average molecular weight; *M_w_*/*M_n_* = molecular weight distribution.

**Table 2:** TGA data under oxidative atmosphere for PCL and PCL-based composites.

Sample	*T*_5%_ (°C)	*T*_50%_ (°C)	R (%)
PCL	378	426	0
PCL–HGM10	384	428	9
PCL–HGM15	388	428	13
PCL–HGM20	390	431	21
PCL–HGM25	391	432	26
PCL–HGMf10	368	420	9
PCL–HGMf20	347	415	20

*T*_5%_ = temperature at which the initial 5% mass loss occurs; *T*_50%_ = temperature at which the initial 50% mass loss occurs; R = residue amount at 700 °C.

**Table 3:** DSC data for PCL and PCL-based composites.

Sample	*T*_c_ (°C)	Δ*H*_c_ (J/g)	*T*_m_ (°C)	Δ*H*_m_ (J/g)	χ_c_ (%)
PCL	31.8	52.7	57.3	52.8	38.0
PCL–HGM10	34.7	47.5	57.9	48.3	38.5
PCL–HGM15	35.3	44.2	57.9	46.0	38.9
PCL–HGM20	35.6	40.7	57.3	42.3	38.0
PCL–HGM25	35.7	38.7	57.4	39.8	38.1
PCL–HGMf10	35.9	47.2	57.8	48.0	38.3
PCL–HGMf20	36.4	42.7	57.8	43.2	38.8

*T*_c_ = crystallization temperature; Δ*H*_c_ = crystallization enthalpy; *T*_m_ = melting temperature; Δ*H*_m_ = melting enthalpy; χ_c_ = crystallinity.

**Table 4:** Uniaxial tensile data for PCL and PCL-based composites.

Sample	*E* (MPa)	σ _yield_ (MPa)	ε _break_ (%)
PCL	241 ± 13	12.8 ± 0.9	1303 ± 40
PCL–HGM10	291 ± 19	8.1 ± 0.8	724 ± 38
PCL–HGM15	343 ± 22	6.4 ± 0.8	682 ± 34
PCL–HGM20	410 ± 20	5.6 ± 0.9	667 ± 14
PCL–HGM25	461 ± 15	5.9 ± 0.4	567 ± 15
PCL–HGMf10	388 ± 21	14.1 ± 0.7	672 ± 34
PCL–HGMf20	513 ± 31	13.8 ± 1.2	384 ± 53

*E* = Young’s modulus; σ_y_ = yield stress; ε_r_ = elongation at break.
